# Disease Burden of Dengue in the Philippines: Adjusting for Underreporting by Comparing Active and Passive Dengue Surveillance in Punta Princesa, Cebu City

**DOI:** 10.4269/ajtmh.16-0488

**Published:** 2017-04-05

**Authors:** Eduardo A. Undurraga, Frances E. Edillo, Jonathan Neil V. Erasmo, Maria Theresa P. Alera, In-Kyu Yoon, Francisco M. Largo, Donald S. Shepard

**Affiliations:** 1 Schneider Institutes for Health Policy, Heller School, Brandeis University, Waltham, MA; 2 Department of Biology, University of San Carlos, Cebu City, Philippines; 3 Department of Health, Cebu City, Philippines; 4 Philippines-AFRIMS Virology Research Unit, Cebu City, Philippines; 5 Armed Forces Research Institute of Medical Sciences, Bangkok, Thailand; 6 Dengue Vaccine Initiative, International Vaccine Institute, Seoul, Republic of Korea; 7 Department of Economics, University of San Carlos, Cebu City, Philippines

## Abstract

Dengue virus (DENV) is a serious threat to public health. Having reliable estimates of the burden of dengue is important to inform policy and research, but surveillance systems are not designed to capture all symptomatic DENV infections. We derived the rate of reporting of dengue by comparing active surveillance of symptomatic DENV infections in a prospective community-based seroepidemiological cohort study (*N* = 1008) of acute febrile illness in Punta Princesa, Cebu City, Philippines, with passive surveillance data from the Cebu City Health Department. Febrile episodes detected in a weekly follow-up of participants were tested for serotype-specific DENV by hemi-nested reverse transcription-polymerase chain reaction (nested RT-PCR) and acute/convalescent blood samples tested by dengue IgM/IgG enzyme immunoassay. We estimated the burden of dengue in the Philippines in disability-adjusted life years (DALYs), and conducted a probabilistic sensitivity analysis using Monte-Carlo simulations to address uncertainty. The results showed a 21% cumulative reporting rate of symptomatic DENV infections, equivalent to an expansion factor of 4.7 (95% certainty level [CL]: 2.2–15.1). Based on surveillance data in the Philippines for 2010–2014, we estimated 794,255 annual dengue episodes (95% CL: 463,000–2,076,000) and a disease burden of 535 (95% CL: 380–994) DALYs per million population using age weights and time discounting and 997 (95% CL: 681–1,871) DALYs per million population without age and time adjustments. Dengue imposes a substantial burden in the Philippines; almost 10 times higher than estimated for rabies, about twice the burden of intestinal fluke infections, and about 10% of the burden of tuberculosis. Our estimates should inform policy makers and raise awareness among the public.

## Introduction

Dengue virus (DENV) is the most important arbovirus among humans. With around half the world population at risk and recent estimates of about 60–100 million symptomatic infections per year,^1^,^2^ DENV imposes a substantial burden to communities and health systems in most tropical and subtropical countries.^3^–^6^ Dengue can be caused by any of four viral serotypes (DENV 1–4); symptoms range from asymptomatic or mild febrile illness to severe dengue and, in some cases, death.^7^,^8^

Dengue is a major public health problem in the Philippines and is endemic in all regions of the country.^9^,^10^ The country's outbreaks are largely seasonal, with most episodes occurring during the wet season (June–February).^11^ The Philippines has made dengue a notifiable disease since 1958, has all four DENV serotypes circulating^9^ and ranks among the countries with the highest number of dengue episodes in southeast Asia.^12^–^14^ On average, 170,503 symptomatic DENV infections and 750 deaths were officially reported to the Philippines Department of Health (DoH) annually from 2010 to 2014, i.e., an incidence of about 178 symptomatic dengue episodes per 100,000 population and a reported case fatality rate of approximately 0.44% (Philippines DoH, unpublished communication, September 2015).^15^ A recent review of the epidemiology of dengue in the Philippines showed that the incidence rate of dengue was highest among children of 5–14 years of age, with over 80% of dengue-related deaths occurring among individuals of less than 20 years of age.^9^

Dengue surveillance in the Philippines depends mostly on disease reporting units (DRUs), which include sentinel hospitals, private clinics, rural health units (RHUs), municipal or city health offices, and human quarantine stations, to report all suspected, probable, and confirmed dengue episodes since 2007 to the Philippines Integrated Disease Surveillance and Response System.^9^,^16^,^17^ The surveillance system largely focuses on hospitalized cases, particularly those with severe symptoms.^10^,^15^ About 93% of all dengue episodes reported in 2010–2014 were hospitalized patients and, of these, half were reported from private facilities.^18^ However, a substantial share of dengue episodes may not be reported, thus hindering estimates of the true burden of dengue in the Philippines.

The complexity of dengue illness limits the accuracy of reporting. Reporting rates vary with severity of symptoms and treatment setting, with more severe, hospitalized, and episodes treated in the public sector more likely to be reported than those less severe, ambulatory, or privately treated.^4^,^12^,^19^–^22^ The severity of DENV infections has been associated with younger age,^23^–^25^ newly introduced serotype,^26^,^27^ secondary infection,^28^–^30^ greater time interval between infections,^23^ and host genotype,^31^,^32^ among other factors that indirectly impact the rate of reporting. Misdiagnosis, particularly in countries with high incidence of other febrile illnesses,^33^–^36^ and underdiagnosis due to limited sensitivity and cost constraints of diagnostics tests may also contribute to underreporting.^37^,^38^ Additional sources of uncertainty in estimates of dengue incidence have been discussed elsewhere,^39^ and several studies have estimated average reporting rates of dengue episodes.^3^,^40^,^41^ Most studies have been limited to cohorts of children and/or adolescents.^40^ Evidence from Puerto Rico and Brazil, both of which have a well-funded surveillance system, suggests that even fatal DENV infections may be underreported.^42^,^43^ These findings, together with the variability in reporting rates shown in previous studies,^21^,^44^,^45^ underscore the need to improve understanding of the relation between passive surveillance and accurate reporting of dengue cases.

Having an accurate estimate of disease incidence and burden of dengue is important to inform decisions about health policy, research, and program impact, based on reliable and comparable measures in time.^39^,^46^ Dengue surveillance systems are essential to estimate disease incidence; however, the sensitivity of surveillance systems is limited. Surveillance systems in most dengue-endemic countries, including the Philippines, are passive, depending on the patient presenting to the professional health sector for treatment and the provider reporting the case to public health authorities. Design and implementation limitations of dengue surveillance systems may hinder accurate estimates of disease burden and challenge evidence-based decision-making, and the need for more effective surveillance systems has long been acknowleged.^39^,^46^–^50^

**Figure 1.**
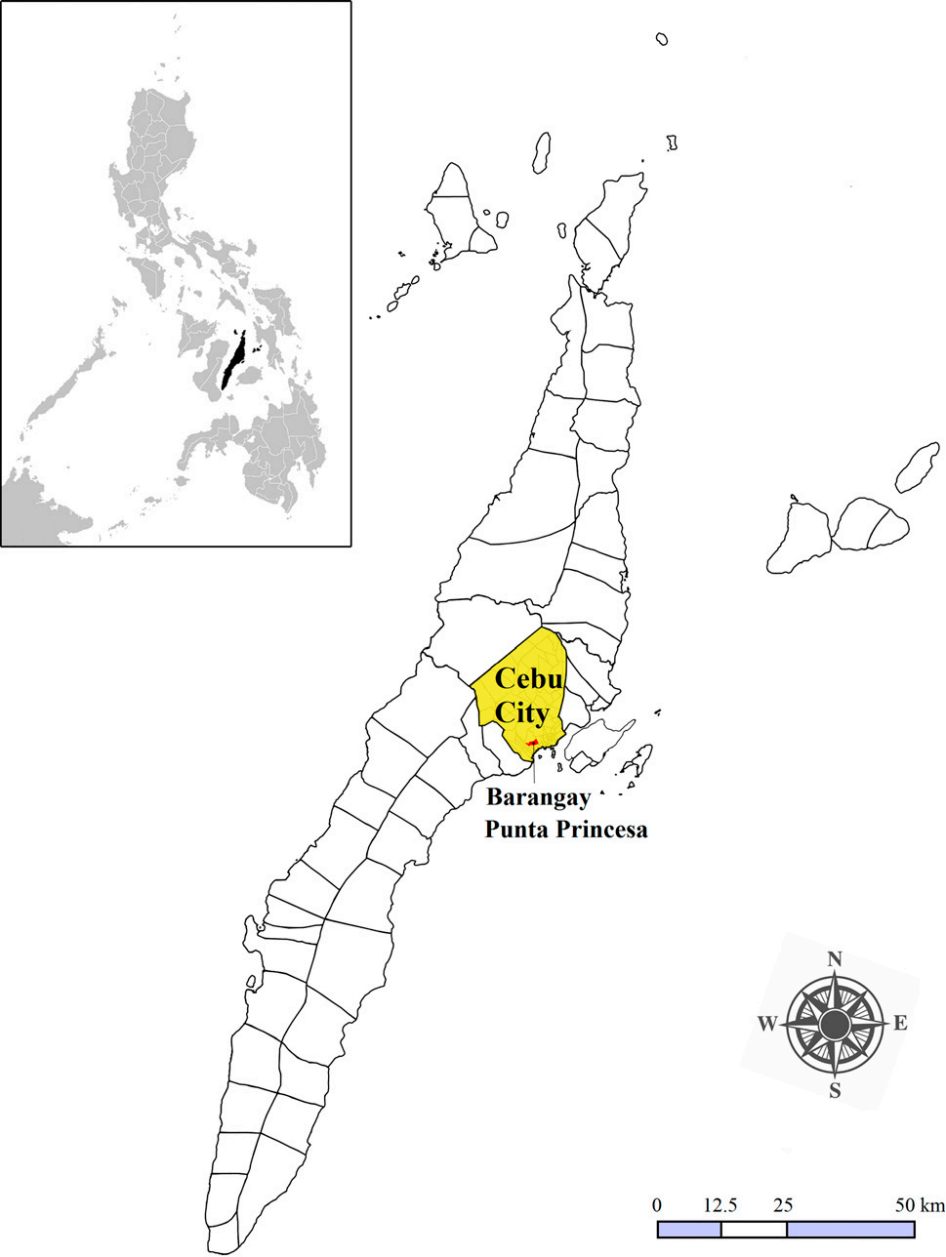
Location of Punta Princesa in Cebu City (shaded), Region VII, Philippines (inset).

Here we estimated the average reporting rate and expansion factor (EF) of dengue episodes in the Philippines comparing active surveillance data of symptomatic DENV infections with cases reported to the surveillance system. Specifically, we compared active surveillance data of symptomatic DENV infections in a prospective community-based seroepidemiological cohort, including children (6 months to 15 years) and adults, in Punta Princesa, Cebu City, Philippines from March 2012 to March 2013 with reported dengue episodes based on passive surveillance data from the Cebu City Health Department (CCHD). Punta Princesa is an urban *barangay* (smallest government unit) located in the South District of the city (Figure 1

), with a population of about 22,400.^53^ Using our adjusted estimate of total dengue episodes, we estimated the disease burden of dengue using disability-adjusted life years (DALYs).

## Materials and Methods

### Passive dengue surveillance.

We obtained the number of reported dengue episodes (April 2012 to March 2013) in Punta Princesa, Cebu City, from the CCHD.^54^,^55^ The case definition used by the CCHD is based on the Manual of Procedures for the Philippine Integrated Disease Surveillance and Response,^56^ which follows the World Health Organization (WHO) 1997 classification of dengue illness (undifferentiated fever, dengue fever, dengue hemorrhagic fever, and dengue shock syndrome).^57^ The Philippines DoH officially updated its dengue classification by levels of severity (dengue without and with warning signs, severe dengue), as recommended by current WHO guidelines,^7^ in its Revised Dengue Clinical Case Management Guidelines.^58^ However, dengue is still reported based on the WHO 1997 classification system because not all hospitals have adopted the new classification scheme. Most reported dengue cases are based on clinical diagnosis and are not laboratory confirmed; a laboratory diagnostic test usually requires out-of-pocket payment by the patient. We obtained census data from the Philippine Statistics Authority^59^ and estimated monthly reported incidence rates of dengue (per 1,000) by dividing the monthly reported dengue cases by the population of Punta Princesa (*N* = 27,303).

### Active dengue surveillance by cohort study.

The Armed Forces Research Institute of Medical Sciences (AFRIMS) and the Philippines AFRIMS Virology Research Unit (PAVRU) conducted a prospective community-based seroepidemiological cohort study in Punta Princesa, Cebu City. The cohort included 1,008 enrolled volunteers. Inclusion criteria for the cohort included the following: 1) male or female ≥ 6 months of age, 2) resident of Punta Princesa, and 3) understood, approved, and signed the written informed consent and/or assent (if children > 12 years of age). The study excluded participants who had suspected active tuberculosis, or lived in the same household as a person with suspected active tuberculosis, to reduce risk to the research staff. Blood samples were collected at baseline and at 12 months. We estimated monthly incidence rates of dengue (per 1,000) in the cohort.

The health status of enrolled cohort participants was monitored weekly through short message service, phone call, and/or home visit by the PAVRU research team, Cebu City. Acute illness in a cohort participant with fever in the previous 7 days or with measured fever (≥ 38°C) was investigated. Participants were clinically assessed at acute, 2-, 5-, and 8-day visits, and a convalescent visit at the third week. Blood samples were collected at the acute and third-week visits from all participants who reported fever in the past 7 days or whose measured fever was ≥ 38°C and were transported to the PAVRU laboratory. Serum aliquots of these blood samples were frozen at ultralow temperatures (−70°C) until ready for further testing. Further details about the cohort and active surveillance have been reported elsewhere.^60^,^61^

### Detection of DENV.

Aliquots of the blood samples of participants with suspected DENV infection were sent for laboratory analysis to AFRIMS. Detection of DENV RNA in the acute blood samples was done by reverse transcription polymerase chain reaction (RT-PCR) following Lanciotti and others^62^ with modifications (see Supplemental Material for further details). Serological testing for evidence of DENV infection was done in the acute phase and third-week blood samples by DENV IgM/IgG ELISA.^60^

### Estimation of EFs.

EFs are used to obtain a more accurate estimate of number of illness episodes and can be estimated as the number of dengue episodes in a specified population and setting divided by the number of episodes reported to the surveillance system (EF = total episodes of dengue/reported episodes). To estimate EFs of dengue episodes in Punta Princesa, we divided monthly incidence rates of laboratory-confirmed dengue episodes from active surveillance (our best estimate of the true incidence of dengue) by the incidence rate of reported dengue episodes based on passive dengue surveillance for Punta Princesa from the CCHD. We estimated the reporting rate (proportion of episodes reported) as the inverse of EF.

### Estimates of the disease burden of dengue.

Despite documented variation of reporting rates in time and location,^21^,^44^,^45^ we used our results to improve estimates of dengue burden in the entire country. We based our burden of disease estimates on average reported nonfatal and fatal dengue cases in the Philippines in 2010–2014, the most recent 5 years of surveillance data available, to provide a more stable estimate of the burden of dengue, considering the substantial annual variation of disease incidence.

We estimated the disease burden of dengue in DALYs, a summary measure of population health that combines morbidity and mortality outcomes.^63^ A DALY is the sum of a measure equivalent to the years of life lost due to disability and a measure of the years lost due to premature death (YLL). DALYs were developed in the early 1990s to compare population health across countries and in time, and the original 1990 Global Burden of Disease (GBD) project used age-weights and time-discounting.^51^,^64^ The definition of DALYs was updated for the GBD 2010 study by Murray and others^63^,^65^,^66^ at the Institute of Health Metrics and Evaluation (IHME), dropping age-weights and time-discounting, which is also the DALY definition currently used by WHO.^67^ To enhance comparability with other studies, we have reported DALYs using both age-weights and time-discounting (hereafter original GBD), and without age-weights and time-discounting (hereafter IHME-GBD).

We obtained duration of illness in ambulatory and hospitalized dengue episodes from a previous study^10^ and the age distribution of fatal (2003–2005) and nonfatal (2000–2009) dengue episodes from the Philippines DoH. We did not use data on duration of illness or age distribution from CCHD, because our objective was to estimate DALYs at the national level. We estimated the years of life lost based on GBD-2010 standard abridged life table for computing years of premature life lost.^65^ We allocated dengue episodes to treatment settings based on the results from a Delphi panel workshop including 34 national and international dengue experts in Cebu City, Philippines, in 2013.^10^ To estimate DALYs using original GBD methodology, we used the same parameters as previous dengue studies,^52^,^68^,^69^ namely, a disability weight of 0.81 (range: 0.6–0.92), age constant of 0.16243, age weight of 0.04, and an annual discount rate of 3%.

### Sensitivity analysis and uncertainty.

Because substantial uncertainty still remained around many of the main parameters in our model, we conducted a probabilistic sensitivity analysis of our estimates based on Monte Carlo simulations. A Monte Carlo simulation consists of running repeated trials, based on random sampling from the probabilistic distribution of the parameters in the model, to obtain the frequency distribution of numbers of dengue episodes and other results of interest. We computed 10,000 Monte Carlo simulations for each parameter, simultaneously varying the following parameters based on ranges and probability distributions in the dengue literature: EF for nonfatal and fatal dengue episodes, proportion of cases hospitalized, average length of stay at the hospital, average number of ambulatory visits prior to hospitalization, average number of visits for ambulatory patients, and disability weights for dengue. To estimate uncertainty for nonfatal EF, we obtained the standard deviation from the sample of monthly estimates of reporting rates and assumed a truncated normal distribution (censored at 5%). For fatal EF, we used a beta-PERT distribution with minimum, mode, and maximum values based on the literature.^42^,^43^ We showed the sensitivity of our estimates to our main model parameters using a tornado diagram.

### Ethics.

The prospective cohort study was approved by the Institutional Review Boards of Vicente Sotto Memorial Medical Center, Cebu City, Philippines, the Walter Reed Army Institute of Research, and the overall dengue burden analysis was approved by the Committee for the Protection of Human Studies in Research at Brandeis University. All participants in the study or their parents (for children under age 18) gave written informed consent and written assent was obtained from children older than age 12.

## Results

### Prospective cohort.

The cohort included 1,008 enrolled volunteers from Punta Princesa, with about 200 per age category at entry (6 months to 5 years, 6–15 years, 16–30 years, 31–50 years, and > 50 years) and a balanced distribution of female and male participants. Table 1 shows the main characteristics of the Punta Princesa, Cebu City, prospective cohort. Of 1,008 participants enrolled, 854 followed all activities during the year of the study following the study protocol. Reasons for not completing all activities included relocation out of the study area, consent withdrawal, lost to follow-up, and developing other health conditions.^58^ No individuals were excluded from enrollment because of their active pulmonary tuberculosis or that of a household member.

### Disease surveillance.

Table 2 compares the incidence rates of symptomatic DENV infections per 1,000 population in Punta Princesa based on active surveillance from the prospective cohort study and from passive surveillance as reported by CCHD. The estimated EFs showed more variation when using monthly incidence compared with cumulative incidence, because with cumulative incidence the sample size increases, providing a more stable estimate, and smooths seasonal differences in reporting rates.^45^

**Figure 2.**
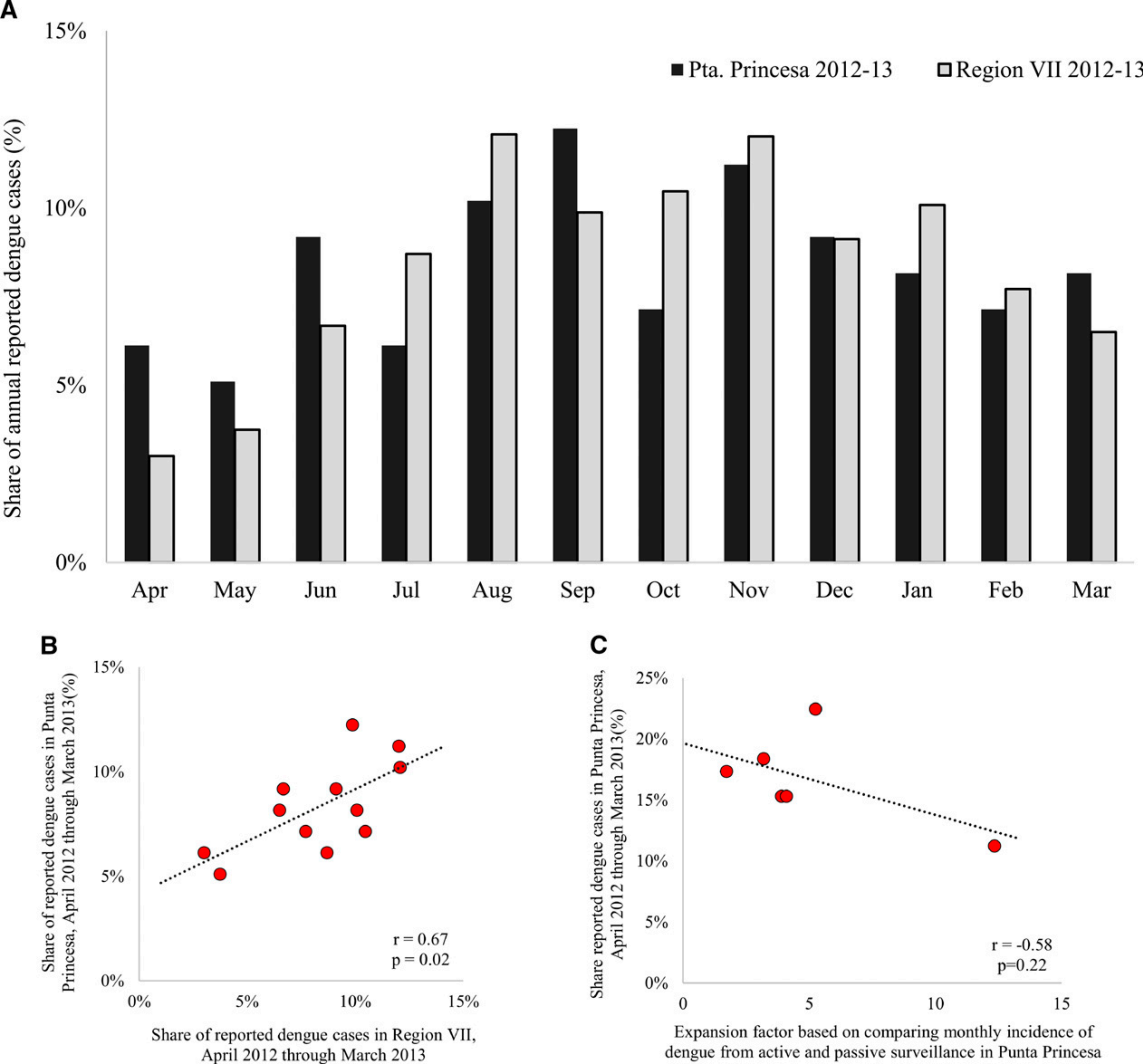
Distribution of reported nonfatal dengue episodes in Punta Princesa and Region VII, Philippines, and expansion factor (EF)-based comparison of monthly incidence of dengue from active and passive surveillance systems in Punta Princesa. (**A**) The distribution of reported dengue episodes by month in Punta Princesa and Region VII, Philippines (April 2012 to March 2013), as a proportion of annual reported dengue episodes. (**B**) The correlation between the monthly distribution of cases in Punta Princesa and Region VII. (**C**) The correlation between the distribution of dengue episodes and the EF based on comparing monthly incidence of dengue from active and passive surveillance systems in Punta Princesa. Pta. denotes Punta.

We next examined whether the reported monthly (April 2012 to March 2013) dengue cases in Punta Princesa (*barangay* level) followed a pattern similar to those reported at the regional administrative level, Central Visayas (Region VII) in the Philippines. Figure 2A

shows these distributions as a proportion of annual reported dengue episodes. Figure 2B shows the correlation between the monthly distribution of cases in Punta Princesa and Central Visayas (*r* = 0.67; *P* = 0.02), which suggests that passive surveillance at both administrative levels was significantly correlated. Figure 2C shows the correlation between the distribution of dengue episodes and the EF based on comparing bimonthly dengue incidence from active and passive surveillance systems in Punta Princesa (*r* = −0.58; *P* = 0.22). Bimonthly EFs were more stable than monthly EFs. We obtained higher EFs during the months when there was higher relative number of dengue episodes (i.e., high season).

### Estimates of the disease burden of dengue.

**Figure 3.**
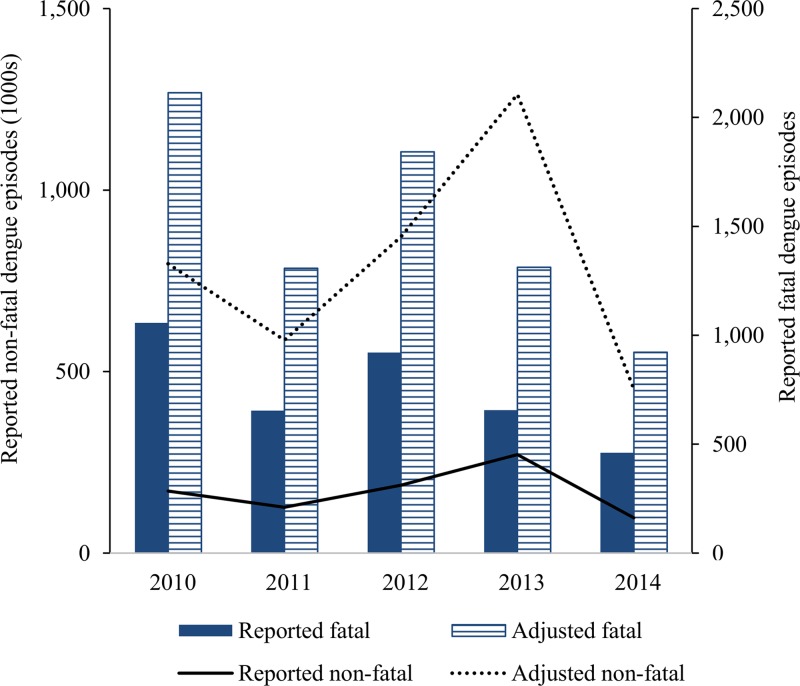
Reported and adjusted dengue episodes in the Philippines, 2010–2014. Adjustment based on expansion factor of 4.7 comparison between active and passive surveillance systems in Punta Princesa.

Dengue incidence varies substantially across years. Our best estimate to adjust for underreporting of dengue episodes in the Philippines based on comparing the cumulative incidence of dengue from active and passive surveillance systems in Punta Princesa is to use an EF = 4.7, that is, for each nonfatal dengue episode reported 4.7 symptomatic nonfatal dengue episodes occur. Even though these data corresponded to a single dengue season, we considered the reporting of symptomatic dengue episodes in 2012 as representative of the latest 5-year available data for two reasons. First, after refining and expanding the surveillance system, the Philippines Integrated Disease Surveillance and Response System remained largely unchanged during 2010–2014.^9^ Second, the number of reported dengue episodes increased in 2010 and subsequently remained consistently high,^9^,^10^ probably largely due to improved reporting, as suggested by a larger increase in dengue cases than in deaths.^9^ Figure 3

shows 2010–2014 reported cases averaging 170,503 nonfatal and 750 fatal. Because the mortality rate for dengue is low, our cohort was not large enough to estimate underreporting of fatal dengue episodes. However, at least two studies performed in Puerto Rico^42^ and Brazil^43^ provide evidence of underreporting of dengue with a range of 2–5 fatal dengue episodes per fatal case reported. We used an EF of two to be conservative (range for sensitivity analysis: 1–5).

Table 3 shows the parameter values, distributions, and data sources used to address uncertainty in our data and to estimate the 95% certainty levels (CL) of our main results. We modeled the variation in reporting rates of nonfatal dengue episodes based on the comparison of monthly incidence rates between passive and active surveillance systems. We estimated an EF of 4.7 (95% CL: 2.2–15.1). We estimated a total of 794,255 annual episodes of dengue (95% CL: 382,161–2,581,385) in the Philippines in 2010–2014. Of these, we estimated a total of 516,266 (95% CL: 228,830–1,630,468) dengue patients were hospitalized annually, based on the treatment setting allocation from a Delphi panel. Last, we estimated a total of 1,500 annual fatal episodes of dengue (95% CL: 907–2,904).

Table 4 shows the main results for disease burden estimates by treatment setting adjusted for underreporting of nonfatal and fatal dengue episodes. We found a substantial disease burden, with 535 (95% CL: 353–988) DALYs per million population using age-weights and time-discounting (original GBD method), and 997 (95% CL: 644–1,838) DALYs per million population without age and time adjustments (IHME-GBD method). The main difference in results between the two methods was driven by YLL, which highlighted the relevance of age-weights and time-discounting for comparability purposes. Dropping time-discounting and age-weights from DALY estimates implied an important shift toward deaths at younger ages and away from valuing more a year of healthy life for those at a more productive age (20–50 years). Most of disease burden of dengue came from YLL (75% original GBD; 87% IHME-GBD).

Figure 4

**Figure 4.**
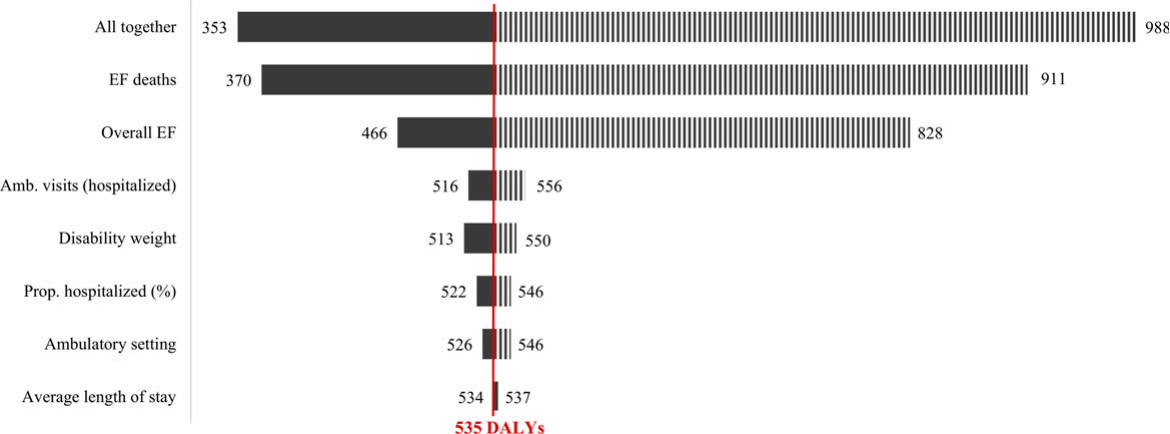
Variability of disease burden estimates in disability-adjusted life years (DALYs) per million population (using the original Global Burden of Disease method), based on the variation of the main parameters in the sensitivity analysis. The point estimate for the disease burden of dengue is shown by the vertical line in the figure at 535 DALYs per million population. All together denotes the simultaneous variation of all the parameters in the model, as shown in Table 3. EF denotes expansion factors, Amb. denotes dengue episodes treated in an ambulatory setting, Prop. hospitalized (%) denotes the proportion of dengue patients that are hospitalized on average, as determined by a Delphi panel,^10^ disability weight refers to the disability weights used for dengue and the corresponding variation.^51^,^52^

shows the main sources of variability in our estimated burden of dengue in DALYs per million population (based on original GBD method, with age-weights and time-discounting). The vertical line at 535 DALYs per million population shows the point estimate for the burden of dengue. The “tornado diagram” shows the 95% CL obtained through the 10,000 Monte Carlo simulations varying each parameter alone and varying all parameters simultaneously (top bar in the diagram). The main source of variation for our estimates came from the estimated EFs, because they determine the estimated incidence of the disease.

## Discussion

Our results confirmed that dengue has been under reported in the Philippines, as previous studies have suggested.^10^,^12^ We found a cumulative reporting rate of 21% of symptomatic DENV infections, equivalent to an EF of 4.7 (95% CL: 2.2–15.1). Because EFs were estimated by comparing dengue incidence rates per person under follow-up in active and passive surveillance, participant attrition should not have affected our main results. Based on surveillance data in the Philippines for 2010–2014, we estimated 794,255 annual dengue episodes (95% CL: 382,161–2,581,385) and a disease burden of 535 (95% CL: 353–988) DALYs per million population using age-weights and time-discounts (original GBD) and 997 (95% CL: 644–1,838) DALYs per million population without age and time adjustments (IHME-GBD).

Our estimated EF was comparable to previous estimates of EFs in the Philippines and also in Central Visayas. Borja and others^70^ found that about 81% of dengue episodes were not reported in Manila, Muntinlupa, Baguio, Iloilo, Cebu, and Davao, which resulted in an overall EF of about 5.3 for these cities. Undurraga and others^12^ estimated that only about 13% of symptomatic dengue episodes in southeast Asia are reported. Using a regression model based on empirical studies from other countries in the region and an index of health quality, that study estimated a reporting rate of 14.3% of all symptomatic DENV infections in the Philippines, or EF of 7.0 (EF = 1/reporting rate). Comparing active surveillance based on preliminary results from this cohort in Punta Princesa, Cebu City, with the CCHD's passive surveillance data from March to October 2012, Edillo and others^10^ derived an empirical reporting rate of 13.3% for the Philippines (EF = 7.2). This preliminary rate is within the 95% CL of the present study. Using data from a dengue vaccine prospective cohort of children (2–14 years of age) in two study centers, Nealon and others^71^ compared incidence densities from active surveillance with incidence rates from the national passive surveillance system and obtained an EF of 11.5 (95% CL: 9.1–14.3). Toan and others^40^ estimated EFs for the Philippines as 15 and 14 episodes of dengue for each reported episode in 2007 and 2010, respectively, by comparing incidence rates from prospective community-based studies with estimated incidence at the country level. Their estimates were based on a follow-up study of young children (aged 2–15 months) in San Pablo, Laguna, in 2007–2008,^72^ and on a community-based enhanced surveillance program of children (2–14 years or age) in various cities in 2010–2011^73^. If these cohorts were done in areas with higher than national average of incidence rates of dengue, these annual EF estimates may be overestimates, but were still within the range we obtained from Punta Princesa. The wide 95% CL for our EF estimates in Punta Princesa reflect the variance in monthly estimates of underreporting, mostly due to the relatively small sample size of our cohort, which had only 15 symptomatic dengue cases.

The results support previous evidence that reporting rates of dengue episodes may vary substantially over time^21^,^44^,^45^ and among locations. These variations may be explained by differential access to health care and health-care quality, providers' attention to dengue, variation in DENV serotypes, patients' health-seeking behavior, and mosquito population densities, among other factors. In the Philippines, dengue surveillance is largely conducted by DRUs; their size, infrastructure, quality of care, and connectivity vary substantially across the country and thus may result in variations in reporting rates by locality. As the DoH in Cebu City has collaborated with local and international partners on dengue research since 2005,^74^ dengue reporting might be better there than in the Philippines overall. It is important to bear in mind that EFs are used to improve estimates of dengue burden. The importance of having exact EF estimates for specific times and locations depends on their application. For example, more refined estimates may be needed to target control strategies most efficiently. Public health officials may need only approximate estimates of disease incidence, however, to support resource allocation between dengue and other conditions.

Our estimate of the annual disease burden of dengue was higher than a previous estimate for 2001–2010 (433 original GBD DALYs per million population),^5^ possibly due to higher incidence of dengue, and comparable to an estimate for 2013 (1,350 IHME-GBD DALYs per million population),^2^ but both estimates fell within our 95% CL. The results suggested that the annual burden of dengue was higher than estimates for other infectious diseases, including rabies (110 and 49 DALYs per million population based on IHME-GBD and original GBD methods, respectively)^75^ and intestinal fluke infections (590 IHME-GBD DALYs per million population in Philippines and Thailand together),^76^ and about 10% the disease burden estimated for tuberculosis (5,350 original GBD DALYs per million population).^77^

Last, even though reporting rates vary by year and geographic area, if we applied the estimated EF to reported episodes of dengue and deaths in the Philippines in 2013, we would obtain a total of 1,264,000 estimated cases of apparent dengue and 1,312 deaths for 2013 (Figure 3). These results are near the lower bound of the total number of dengue episodes estimated for the Philippines in 2013 by Stanaway and others^2^ (3.9 million 95% CL: 1.4–8.6) and are comparable to their estimated dengue deaths (1,210 95% CL: 450–1,612). The nearly 820,000 estimated number of hospitalized patients for 2013 based on a Delphi panel in the Philippines^10^ was about twice the 386,000 inpatient episodes estimated in Shepard and others^6^ for 2013, based on extrapolations from other studies.

The relatively limited study length and geographic area of the study restricted our ability to extrapolate results to other years and regions. Dengue cases in Punta Princesa, Cebu City, represented 0.06% of the total dengue cases reported in the Philippines by DoH 2012, or 0.09% relative to the mean number of cases (2008–2012) in the entire country, which has about 40,000 barangays. As discussed above, reporting rates of dengue vary temporally and geographically due to variation in dengue epidemiology, surveillance practices, demographics, health-care infrastructure, and access, all of which may affect the accuracy of our estimates. We would encourage initiating additional sites with active surveillance, particularly in locations that have not participated in previous research. Comparisons between active and passive surveillance in such sites should result in more nationally representative estimates of EFs. Such studies could rely on community health workers for active surveillance of febrile illness followed by diagnostic testing, particularly as dual (NS1 and IgG/IgM) rapid diagnostic tests become more accurate, easier to use, and less expensive. Such studies would benefit participants through improved access to dengue diagnosis and treatment and policy makers through better epidemiological data.

However, the fact that previous studies have shown comparable results underscores the validity of our main conclusions. Despite active surveillance, some dengue illnesses may still have gone undetected, particularly milder episodes. Because reporting of dengue varies by severity and treatment setting, it would have been helpful to distinguish underreporting of inpatient and outpatient episodes separately to obtain a more accurate estimate of disease burden. Unfortunately, estimating an EF by treatment setting requires a much larger study cohort. To strengthen evidence about underreporting, it would have been ideal to compare whether specific patients detected in the active surveillance were also reported in the CCHD passive surveillance; unfortunately, we lacked the data to do so due to privacy protections within each data source. Another limitation includes the reliance on expert opinion to allocate dengue cases by treatment setting.^10^ Finally, our estimates of disease burden did not include persistent symptoms, such as fatigue, asthenia, depression, and weight loss, that have been associated with DENV infection,^8^,^78^ as acknowledged by the WHO since 1997.^57^ Persistent symptoms may represent about a 40% increase in disease burden estimates over those from acute impacts.^78^

## Conclusions

Our results provided evidence that a substantial number of symptomatic DENV infections have not been accounted for in routine reporting in the Philippines, as has been empirically found elsewhere. There are several ongoing efforts to control DENV transmission, including vaccines,^79^–^81^ antiviral drugs,^82^–^84^ and various strategies of vector control.^85^–^88^ The Philippines has a high dengue incidence and has already initiated a school-based dengue vaccination program in Manila.^89^ These estimates of the disease burden of dengue should help inform and refine policy decisions and increase understanding of dengue among the public.

## Supplementary Material

Supplemental Datas.

## Figures and Tables

**Table 1:** Characteristics of the prospective cohort in Punta Princesa, Cebu City, Philippines, March 2012 to March 2013

Characteristic	*N* (%)
Enrolled participants	1,008 (100.0)
Participants who completed study*	854 (84.7)
Females at enrollment	508 (50.4)
Participants by age group: (enrolled/completed)	
6 months to 5 years	203 (20.2)/148 (17.4)
6–15 years	201 (20.0)/184 (21.6)
16–30 years	200 (19.9)/168 (19.7)
31–50 years	204 (20.2)/172 (20.1)
> 50 years	200 (19.8)/182 (21.3)
Participant's household size at enrollment	
1	16 (1.6)
2–3	207 (20.5)
4–6	526 (52.2)
7–10	237 (23.5)
> 10	22 (2.2)
Number of children < 16 years in household at enrollment	
0	199 (19.7)
1	231 (22.9)
2	229 (22.7)
3	180 (17.9)
> 3	169 (16.8)

* Participants who completed all study activities considered in the study protocol at 12 months including enrollment and 12-month blood collections.

**Table 2:** Symptomatic dengue infection incidence rates per 1,000 population in Punta Princesa from active surveillance in the prospective cohort and from passive surveillance as reported by the CCHD

Month	Punta Princesa cohort (*n*)	Incidence rate per 1,000 pop.		Expansion factors as a function of:	
		Pta. Princesa cohort	CCHD*	Monthly incidence (per 1,000 pop.)	Cumulative incidence† (per 1,000 pop.)
April 2012	581	1.72	0.22	7.8	7.8
May 2012	922	3.25	0.18	17.8	12.3
June 2012	922	0.00	0.33	0.0	6.8
July 2012	932	2.15	0.22	9.8	7.5
August 2012	932	3.22	0.37	8.8	7.8
September 2012	988	1.01	0.44	2.3	6.5
October 2012	968	0.00	0.26	0.0	5.6
November 2012	948	2.11	0.40	5.2	5.6
December 2012	941	0.00	0.33	0.0	4.9
January 2013	931	1.07	0.29	3.7	4.8
February 2013	923	1.08	0.26	4.2	4.7
March 2013	908	1.10	0.29	3.8	4.7

CCCH = Cebu City Health Department; pop. = population; Pta. Princesa = Punta Princesa cohort study.

* CCHD rate shows the incidence rate per 1,000 population of symptomatic dengue infections in Pta. Princesa as reported through passive surveillance.

† Cumulative reflects average since April 2012.

**Table 3:** Parameters values, probabilistic distributions, and sources of data used in the probabilistic sensitivity analysis

Item	Best	Parameters	Values	Distribution	Source
Reporting rate for nonfatal dengue (%)	21	(μ, σ)	(21, 12)	Normal*	Pta. Princesa active and passive surveillance
Expansion factor for fatal dengue	2.0	(min, mode, max)	(1.0, 2.0, 5.0)	Beta-PERT†	Tomashek and others^42^; Pamplona and others^43^
Percentage of cases hospitalized (%)	65	(min, mode, max) 40	(40, 65, 80)	Beta-PERT	Delphi panel‡^10^
Length of stay in hospital (days)	4.21	(min, max)	(4.02, 4.38)	Uniform	Edillo and others^10^
Ambulatory visits before hosp.(*n*)	4.6	(min, mode, max)	(2.3, 4.6, 6.9)	Beta-PERT	Edillo and others^10^
Visits ambulatory treatment	4.2	(min, mode, max)	(2.1, 4.2, 6.3)	Beta-PERT	Edillo and others^10^
Disability weights DALYs	0.81	(min, mode, max)	(0.60, 0.81, 0.92)	Beta-PERT	Meltzer and others^52^; Murray 1994^51^

DALY = disability-adjusted life year; hosp. = hospital; max. = maximum; min. = minimum; *n* = number; Pta. Princesa = Punta Princesa cohort study.

* The standard deviation was obtained from the sample of monthly estimates of reporting rates.

† The Beta-PERT is a specific form of the beta distribution in which the mean and standard deviation are estimated as a function of expert's assessment of minimum, maximum, and mode values (PERT approximation). We used a scale parameter λ = 4 for the distribution.

‡ The allocation of dengue episodes to treatment settings was based on the results from a Delphi panel workshop conducted in 2013 in Cebu City, the Philippines, which included 34 national and international experts.^10^

**Table 4:** Annual disease burden of nonfatal and fatal dengue in the Philippines (2010–2014)

Indicator (per million population)	Original GBD*	IHME-GBD†
YLD—ambulatory	27.0	27.0
95% CL	10–94	10–94
YLD—hospitalized	105.4	105.1
95% CL	44–337	42–330
YLL‡	402.3	865.2
95% CL	247–773	530–1,663
DALYs	534.8	997.3
95% CL	353–988	644–1,838

CL = certainty level; DALYs = disability-adjusted life years; YLD = years lost due to disability; YLL = years of life lost due to premature death.

* Original Global Burden of Disease (GBD) refers to the original definition of DALYs proposed by Murray in 1994,^51^ and subsequently used by Global Burden of Disease studies conducted by the World Health Organization. We used the same parameters as in previous studies^52^,^68^,^69^ for comparability.

† IHME-GBD refers to an updated definition of DALYs adopted by Murray and others at the Institute of Health Metrics and Evaluation (IHME) for the GBD 2010 study,^65^ where age-weighs and time-discounts were dropped from disease burden estimates. Without age or time discounts, the estimates are YLD = incidence × duration × disability weight; and YLL = incidence × year of life lost due to premature death. The full equation and rationale for original GBD are described elsewhere.^54^

‡ We estimated the years of premature life lost based on GBD-2010 standard abridged life table for computing years of premature life lost.^65^
