# Supplementary material for: Disease Burden of Dengue in the Philippines: Adjusting for Underreporting by Comparing Active and Passive Dengue Surveillance in Punta Princesa, Cebu City
Source: Am J Trop Med Hyg. 2017 Apr 5;96(4):887–98. doi: 10.4269/ajtmh.16-0488 (PMC5392638; doi:10.4269/ajtmh.16-0488)
Supplement: Supplementary file 1 [file SD6.pdf]

Detection of dengue virus. Aliquots of the blood samples of participants with suspected dengue virus (DENV) infection were placed in cryoboxes with dry ice and shipped in a bigger box by a courier to the Armed Forces Research Institute of Medical Sciences, Bangkok, Thailand, in a quarterly basis.

Detection of DENV serotypes was done by reverse transcription polymerase chain reaction (RT-PCR) following Lanciotti and others[1] with modifications. Briefly, the RT-PCR master mix (45 µL reaction) was composed of DNase/RNase free water (30.65 µL), 10× buffer (5 µL) with 25 mM $MgCl_2$ (3 µL), 10 mM dNTPs (1 µL), each of D1 and D2 primers (10 pmol/µL; 1.25 µL), 0.1M dithiothreitol (DTT; 2.5 µL), avian myeloblastosis virus reverse transcriptase (AMV-RT; 10 U/µL; 0.10 µL), and ampliTaq (5 U/µL; 0.25 µL). The master mix was kept at −20°C ± 5°C until use. RT-PCR conditions were at 42°C (60 minutes) and 35 PCR cycles at 94°C (30 seconds), 53°C or 55°C (1 minute), and 72°C (2 minutes). Incubation was at 4°C–10°C. The RT-PCR product (2 µL) was diluted at 1:50 with DNase/RNase-free water (98 µL) for nested-PCR to determine the DENV serotypes. Nested-PCR components per reaction were DNase/RNase free water (13.75 µL), ampli*Taq* (5 U/µL; 0.125 µL), 10× buffer (2.5 µL), 25 mM $MgCl_2$ (2.5 µL), 10 mM dNTPs (0.5 µL), and 0.625 µL per 10 pmol/µL of each of the primers (D1, TS1, TS2, TS3, and TS4). Nested-PCR conditions were the same as in RT-PCR except for 25 PCR cycles. Agarose gel (1.5% w/v) with buffer (1× tris-borate-ethylenediaminetetraacetic acid [TBE]) was used in gel electrophoresis. The gel was mounted in the electrophoresis chamber containing 1× TBE buffer with 0.2 µg/mL ethidium bromide. The PCR (RT or nested) product (9 µL) and 10× gel loading buffer (1 µL) were mixed in 0.5-mL microcentrifuge tubes. The samples were loaded in the middle and flanked by the positive RT-PCR control (left side) and positive extraction control (right side). DENV control strains for serotyping consisted of DENV-1 (Hawaii strain), DENV-2 (New Guinea C strain), DENV-3 (H87 strain), and DENV-4 (814669 strain). The positive extraction control was made of 2.5 or 5 µL of RNA suspension obtained from each DENV control strain and one reaction of RT-PCR mixture. The positive RT-PCR control was made of RNA suspension of the DENV 1–4 mixture, which had been previously tested positive by PCR. The negative extraction control was made of 2.5 or 5 µL of 140 µL RNase-free water extracted along with samples mixed in one reaction of RT-PCR mixture (total volume = 25 or 50 µL), whereas negative RT-PCR control was composed of 2.5 or 5 µL of RNase-free water added in one reaction of RT-PCR mixture (total volume = 25 or 50 µL). Both of these negative controls were loaded next to the positive extraction control. Repeat assays of blood samples were performed if DNA bands had unclear diagnostic size for each DENV serotype, or with multiple DNA bands of DENV serotypes, or if RT-PCR showed positive result but nested-PCR showed negative result.

SUPPLEMENTAL REFERENCE

1. Lanciotti RS, Calisher CH, Gubler DJ, Chang G-J, Vorndam AV, 1992. Rapid detection and typing of dengue viruses from clinical samples by using reverse transcriptase-polymerase chain reaction. *J Clin Microbiol 30:* 545–551.
